# The Alpha 7 Nicotinic Acetylcholine Receptor Does Not Affect Neonatal Brain Injury

**DOI:** 10.3390/biomedicines10082023

**Published:** 2022-08-19

**Authors:** Maria E. Hammarlund, C. Joakim Ek, Sukaina Akar, Alma Karlsson, Bagmi Pattanaik, Filip Mjörnstedt, Pernilla Svedin, Maryam Ardalan, Eridan Rocha-Ferreira, Carina Mallard, Maria E. Johansson

**Affiliations:** 1 Departent of Physiology, Institute of Nefigureuroscience and Physiology, Sahlgrenska Academy, University of Gothenburg, 405 30 Gothenburg, Sweden; 2 Department of Obstetrics and Gynecology, Institute of Clinical Sciences, Sahlgrenska Academy, University of Gothenburg, 405 30 Gothenburg, Sweden

**Keywords:** hypoxia-ischemia, neonatal mice, brain injury, alpha 7 nicotinic acetylcholine receptor, inflammation

## Abstract

Inflammation plays a central role in the development of neonatal brain injury. The alpha 7 nicotinic acetylcholine receptor (α7nAChR) can modulate inflammation and has shown promising results as a treatment target in rodent models of adult brain injury. However, little is known about the role of the α7nAChR in neonatal brain injury. Hypoxic-ischemic (HI) brain injury was induced in male and female C57BL/6 mice, α7nAChR knock-out (KO) mice and their littermate controls on postnatal day (PND) 9–10. C57BL/6 pups received i.p. injections of α7nAChR agonist PHA 568487 (8 mg/kg) or saline once daily, with the first dose given directly after HI. Caspase-3 activity and cytokine mRNA expression in the brain was analyzed 24 h after HI. Motor function was assessed 24 and 48 h after HI, and immunohistochemistry was used to assess tissue loss at 24 h and 7 days after HI and microglial activation 7 days after HI. Activation of α7nAChR with the agonist PHA 568487 significantly decreased CCL2/MCP-1, CCL5/RANTES and IL-6 gene expression in the injured brain hemisphere 24 h after HI compared with saline controls in male, but not female, pups. However, α7nAChR activation did not alter caspase-3 activity and TNFα, IL-1β and CD68 mRNA expression. Furthermore, agonist treatment did not affect motor function (24 or 48 h), neuronal tissue loss (24 h or 7 days) or microglia activation (7 days) after HI in either sex. Knock-out of α7nAChR did not influence neuronal tissue loss 7 days after HI. In conclusion, targeting the α7nAChR in neonatal brain injury shows some effect on dampening acute inflammatory responses in male pups. However, this does not lead to an effect on overall injury outcome.

## 1. Introduction

Hypoxic-ischemic encephalopathy (HIE) affects 1 to 8 in every 1000 newborns in high income countries, and as many as 26 in 1000 in low and middle income countries [1]. Brain injuries observed with HIE are associated with decreased supply of blood and oxygen to the brain. This can give rise to a number of complications, such as cerebral palsy (CP), mental retardation and death [2]. In the last decade, hypothermic treatment has been introduced, which decreases the mortality and risk for severe complications [3,4,5]. However only one in eight newborns respond to this treatment [5], and it is therefore of great importance to find adjuvant therapeutic strategies. 

Inflammation plays an important role in the development of neonatal brain injury [6]. Neonatal HIE activates an inflammatory response [7,8], and anti-inflammatory treatments have been proposed as a potential therapeutic strategy [9]. Microglia is the main immune cell of the nervous system and contributes to homeostasis and immunosurveillance. Upon pathological conditions in the brain, such as HIE, microglia rapidly become activated and turn into a more amoeboid phenotype, presenting antigens, secreting cytokines and other inflammatory mediators [10].

The alpha 7 nicotinic acetylcholine receptor (α7nAChR) is a homopentameric nicotinic receptor consisting of five α7 subunits. In the central nervous system, it is expressed by neurons as well as glial cells and is involved in cognition and memory. Several studies have shown that the receptor display neuroprotective properties [11,12], while decreased receptor expression has been linked to neurological disorders, such as Alzheimer’s diseases and schizophrenia [13]. Despite being classified as a neuronal nicotinic receptor, α7nAChR is also expressed on immune cells, where it acts as a key mediator in the cholinergic anti-inflammatory pathway and can modulate immune responses [14]. Due to this dual effect of anti-inflammation and neuroprotection, it has been proposed as a possible target for stroke treatment in adults, and several studies have shown promising results for agonist treatment in male rodents [15,16]. However, a study in a murine model of preterm excitotoxic brain injury showed that α7nAChR activation worsened the injury, while α7nAChR ablation was neuroprotective [17]. While the number of publications of α7nAChR treatment in adult brain injury is increasing, knowledge of α7nAChR in neonatal brain injury remains sparse. To the best of our knowledge, all studies of α7nAChR treatment in adult brain injury [15,16,18,19,20,21] have so far been carried out in male subjects only, or in the case of the preterm excitotoxic injury [17], in mixed sex cohorts. In this study, we investigated the effect of α7nAChR stimulation and ablation on inflammation and injury in a model of neonatal hypoxic-ischemic (HI) brain injury in male and female mice separately.

## 2. Methods

### 2.1. Experimental Design

**Figure 1:**
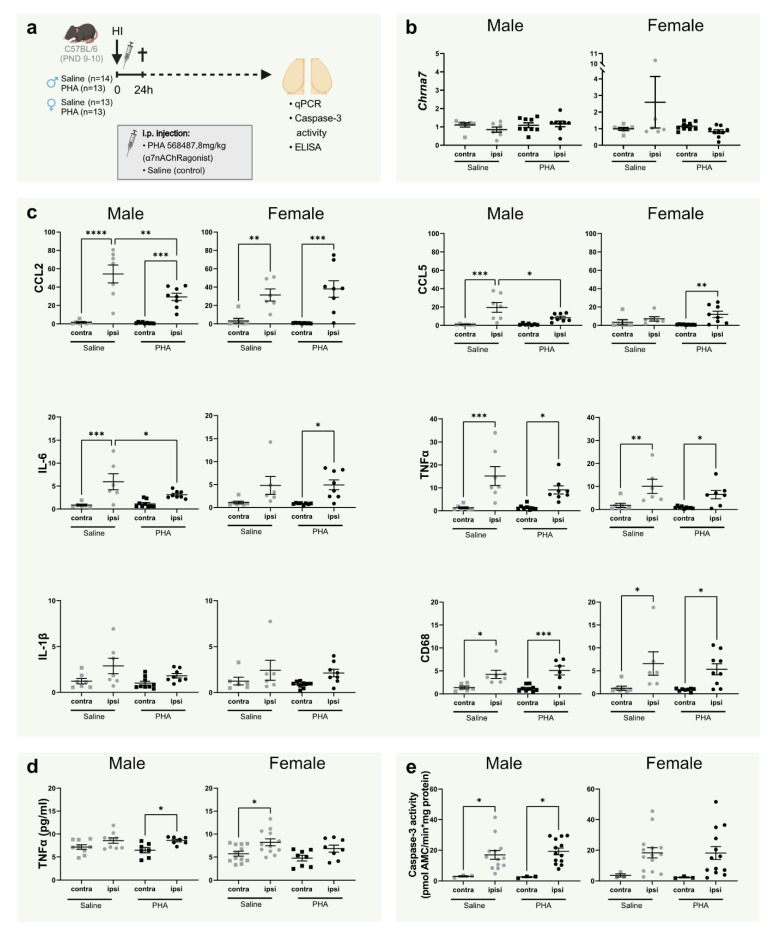
α7nAChR activation decreases CCL2/MCP-1, CCL5/RANTES and IL-6 gene expression in the brain in male mice 24 h after neonatal hypoxia-ischemia. (**a**) Experimental timeline. Male (**left**; *n* = 13–14 per group) and female (**right**; *n* = 13 per group) C57BL/6 pups were subject to HI on PND9–10 immediately followed by i.p. injections with either α7nAChR agonist PHA 568487 (8 mg/kg, black) or saline (gray) and then sacrificed 24 h after HI. (**b**,**c**) mRNA expression of cerebral *Chrna7* (**b**) and CCL2/MCP-1, CCL5/RANTES, IL-6, TNFα, IL-1β and CD68 (**c**) in the injured (ipsi) and non-injured (contra) hemisphere 24 h after HI, analyzed with qPCR. Data are expressed as 2^−ΔΔCT^, where the average of the contralateral hemisphere of the saline group was used as the control (*n* = 6–8/group). (**d**) Protein levels of TNFα measured with ELISA. (**e**) Caspase-3 activity in the brain 24 h after HI in the injured (ipsi) and non-injured (contra) hemisphere. Data were analyzed with one-way ANOVA followed by Sidak’s multiple comparison test and are expressed as mean ± SEM. *p* < 0.05 was considered significant. * *p* < 0.05, ** *p* < 0.01, *** *p* < 0.001, **** *p* < 0.0001.

**Figure 2:**
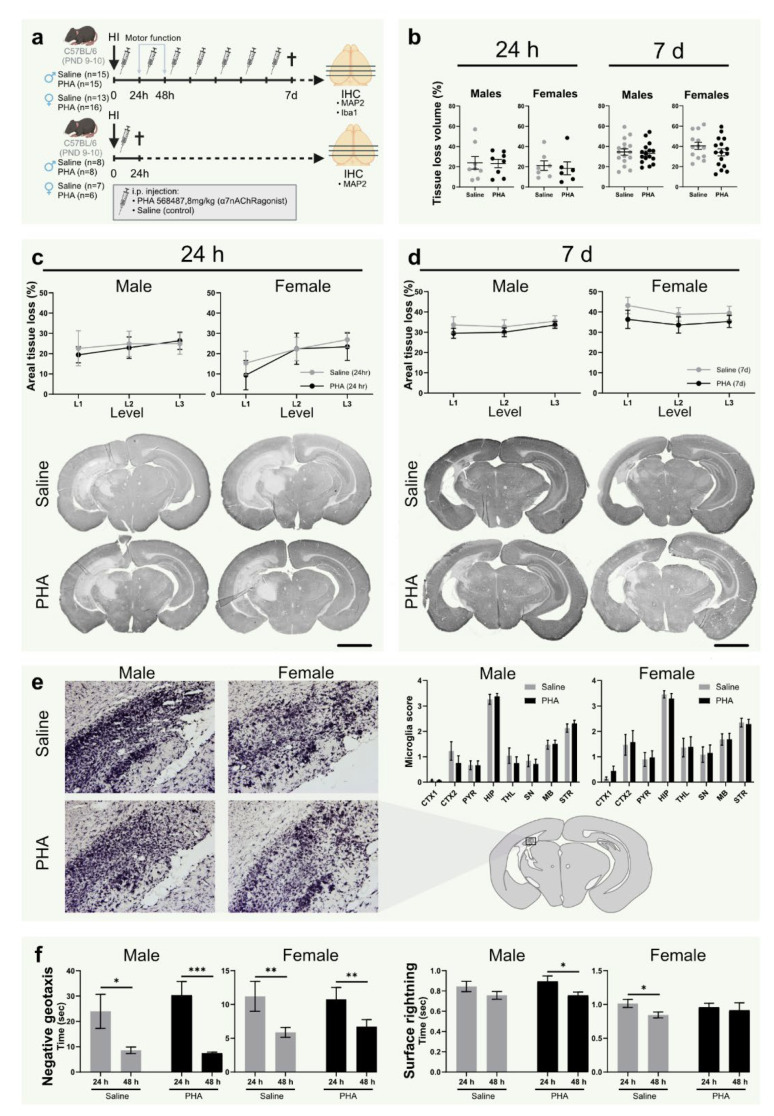
α7nAChR activation does not affect tissue loss, microglial activation or motor function in neonatal hypoxic-ischemic brain injury. (**a**) Experimental timeline. Male (**left**) and female (**right**) pups were subject to hypoxia ischemia (HI) on postnatal day (PND) 9–10 and received i.p. injections once daily with either α7nAChR agonist PHA 568487 (8 mg/kg; black) or saline (gray) until sacrifice 24 h (*n* = 6–8 per group) or 7 days (*n* = 13–16 per group) after HI. (**b**) Tissue loss volume (%) in the brain 24 h (**left**) and 7 d (**right**) after HI. (**c**,**d**) Areal tissue loss (%) at different coronal levels (caudal-rostral direction) of the brain 24 h (**c**) and 7 d (**d**) after HI. Representative micrographs (1.25×) of coronal sections (level 1) stained with the neuron dendrite marker MAP2. (**e**) Representative micrographs (20×) of hippocampus in the injured hemisphere 7 d after HI in sections (level 2) stained with the microglia/monocyte marker Iba-1. Bar graphs show microglial activation score in different regions of injured hemisphere 7 days after HI. (**f**) Recorded time in negative geotaxis (**left**) and surface righting test (**right**) 24 and 48 h after HI. HI: hypoxia-ischemia; PND: postnatal day; CTX1–2: cortex, PYR: pyriform cortex, SN: substantia nigra, MB: midbrain, HIP: hippocampus, TH: thalamus, STR: striatum. Data in (**c**,**d**) is analyzed with two-way analysis of variance with experimental day as nuisance factor and expressed as estimated marginal means ± SEM. Data in (**e**,**f**) are expressed as mean ± SEM. *p* < 0.05 was considered significant. * *p* < 0.05, ** *p* < 0.01, *** *p* < 0.001. Scale bar: 2 mm.

**Figure 3:**
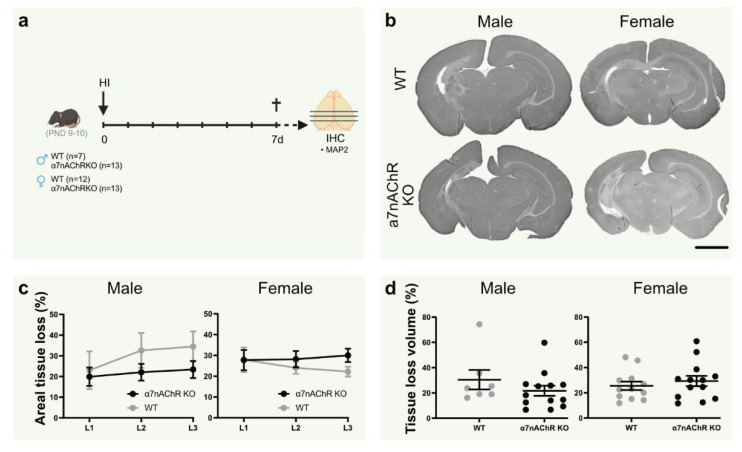
Knock-out of α7nAChR does not affect tissue loss in neonatal hypoxic-ischemic brain injury. (**a**) Experimental timeline. Male (**left**; *n* = 7–13 per group) and female (**right**; *n* = 12–13 per group) α7nAChR knock-out (KO; black) and wild-type (WT) littermate control (gray) pups were subject to hypoxia-ischemia (HI) on postnatal day (PND) 9–10, and sacrificed after 7 days. (**b**) Representative micrographs (1.25×) of coronal sections (level 1) stained with the neuron dendrite marker MAP2. (**c**) Areal tissue loss (%) at coronal levels (caudal-rostral direction) of the brain 7 d after HI. (**d**) Tissue loss volume (%) in the brain 7 d after HI. Data are expressed as mean ± SEM. *p* < 0.05 was considered significant. Scale bar: 2 mm.

To investigate the role of the alpha 7 nicotinic acetylcholine receptor (α7nAChR) in neonatal hypoxic-ischemic (HI) brain injury, male and female mice pups were subject to hypoxic-ischemic brain injury on postnatal day (PND) 9–10. To investigate the effect of α7nAChR activation, C57BL/6 pups were injected intraperitoneal (i.p.) with either the α7nAChR agonist PHA 568487 (8 mg/kg) or corresponding volume of saline (0.9%) once daily until sacrifice (Figure 1a and Figure 2a), and α7nAChR knock-out mice were used to study if absence of α7nAChR had an effect on neonatal HI brain injury (Figure 3a). For analysis of acute immune responses, apoptosis and tissue loss, brains were collected from pups sacrificed 24 h after HI (PND10–11). For analysis of neuronal tissue loss and microglial activation, brains were collected from pups sacrificed 7 days after HI (PND16–17). Agonist- and saline-treated mice were tested for motor function 24 and 48 h after HI.

### 2.2. In Vivo Procedures and Treatment

#### 2.2.1. Animals

C57BL/6 mice were bred in-house with breeding pairs originally purchased from Janvier Labs (Janvier Labs, Le Genest-Saint-Isle, France). Alpha 7 nicotinic acetylcholine receptor heterozygote mice (B6.129S7-Chrna7 tm1Bay/J, α7nAChR^+/−^) were obtained from the Jackson Laboratory (Bar Harbor, ME, USA) and bred in-house. α7nAChR^−/−^ and littermate α7nAChR^+/+^ mice were used in the current study. Genotypes were confirmed using the genotyping protocol available from Jackson Laboratory, including primers for both knock-outs and wild types. Animals were kept on a 12 h/12 h light/dark cycle with unlimited access to laboratory chow and water. All experiments in this study were in compliance with ethical permission approved by the Regional Animal Ethics Committee of Gothenburg, in accordance with the European Communities Council Directives of 22 September 2010 (2010/63/EU).

#### 2.2.2. Hypoxia-Ischemia

Neonatal hypoxic-ischemic (HI) brain injury was induced as previously described [22,23]. In brief, male and female pups (PND9–10) were anesthetized using isoflurane 5% (Attane vet, VM Pharma, Stockholm, Sweden). The left common carotid artery was ligated using a silk 7/0 suture and the incision was closed using Vetbond tissue adhesive (3M, Saint Paul, MN, USA). Following the surgery, which lasted less than 5 min, pups were returned to the dam for 1 h of recovery. The pups were then placed in a chamber kept at 36 °C (10 min air, 50 min 10% O_2_, 10 min air), after which they were returned to their home cage. PND9–10 was chosen as brain development in mice at this age corresponds approximately to that of human term infant [24].

#### 2.2.3. Treatment

The α7nAChR selective agonist PHA 568487 [25] (Tocris, Abingdon, UK) was used to study the effect of α7nAChR stimulation on neonatal HI brain injury. C57BL/6 pups were injected with i.p. PHA 568487 (8 mg/kg) or saline (0.9%, Apoteket AB, Solna, Sweden) immediately following HI at PND 9–10, and then once daily for 1 week until sacrifice.

### 2.3. Behavioral Assessments of Motor Function

Negative geotaxis and surface righting tests [26] were used to investigate the effect of α7nAChR stimulation on motor function after HI. Assessments were performed 24 and 48 h after HI. Replicates of surface righting and negative geotaxis were altered, with 5 min of rest given between each replicate. The surface righting test assesses the pups’ ability to right themselves from their back to regular standing position. Pups were placed on their back on a flat surface and the time and direction (left/right) for righting was recorded. Measurements were recorded in triplicates for each time point, and the mean value for each time point was used for further analysis. The negative geotaxis test assesses the pup’s ability to right themselves on an angled surface (approximately 45°) as described previously [26]. The time was measured for them to turn around to upward facing position. Measurements were run in triplicates for each timepoint, and the value for best performance was used for further analysis.

### 2.4. Sacrifice and Tissue Harvest

Pups were sacrificed 24 h (protein, RNA analysis and immunohistochemistry) or seven days (immunohistochemistry) after HI by intraperitoneal injection of an overdose of pentobarbital (APL, Stockholm, Sweden) (Figure 1a, Figure 2a and Figure 3a). Mice were immediately perfused intracardially with saline to clear blood vessels from blood, and mice used for immunohistochemistry were further perfused with Histofix (Histolab, Askim Sweden). Brain hemispheres used for RNA and protein analysis were isolated in cryo tubes and immediately frozen in liquid nitrogen and stored at −80 °C. Brains used for immunohistochemistry were carefully dissected out and immersion fixed in Histofix until dehydration.

### 2.5. Protein and RNA Analysis

Frozen hemispheres collected 24 h after HI were lightly thawed and homogenized in 500 µL of cold RNAse-free PBS using a hand homogenizer. Brain homogenate was then further processed for protein measurement or immediately frozen on dry ice and stored at −80 °C until RNA preparation. Five hundred microliters of homogenization buffer (1% protease inhibitor cocktail, P8340; 10 mM EDTA; 2% Triton X-100; in PBS) was added to the remaining homogenate, sonicated, centrifuged (10,000× *g*, 4 °C, 10 min) and the supernatant stored at −80 °C until protein analysis.

### 2.6. Caspase-3 Activity Assay

Activity of caspase-3 was measured to evaluate the effect of α7nAChR activation on apoptosis after HI as previously described [27]. In brief, cleavage of the caspase substrate Ac-DEVD-AMC (#SAP3171-v, Peptide Institute, Osaka, Japan) was measured in homogenate supernatants of cerebral hemispheres collected 24 h after HI. Samples were analyzed in a 96-well plate with a Spectramax Gemini plate reader at an excitation wavelength of 380 nm and emission wavelength of 460 nm. The maximal cleavage rate was normalized to protein concentration from BCA assay measurement and expressed as pmol AMC/mg protein and minute.

### 2.7. ELISA

ELISA was used to measure the protein level of the pro-inflammatory cytokine TNF-α in homogenate supernatants from cerebral hemispheres collected 24 h after HI. The ELISA MAX™ Standard Set Mouse TNF-α kit (BioLegend, San Diego, CA, USA) was used according to the manufacturer’s protocol.

### 2.8. QPCR

RNA was extracted from cerebral hemispheres homogenized in RNAse-free PBS using the RNeasy Lipid Tissue Mini Kit (Qiagen GmbH, Hilden, Germany), and the RNA concentration was measured with NanoDrop (Thermo Fisher Scientific, Waltham, MA, USA). QuantiTect Reverse Transcription Kits (Qiagen GmbH, Hilden, Germany) were utilized for cDNA synthesis according to manufacturer’s protocol. cDNA was analyzed with real-time PCR on a LightCycler 480 (Roche Diagnostics GmbH, Mannheim, Germany) as previously described [28]. Melting curves were analyzed for confirmation of single PCR products. Samples were run in duplicates, and samples with an intersample difference of >0.75 cycle were excluded. Primers for α7nAChR encoding gene, *Chrna7* (Forward: 5′ to 3′ GCATGAAGAGGCCGGGAGAGGACAAG, Reverse: 5′ to 3′ GTGTGTGGTCGTTTGGCCTGCTCCC; Invitrogen, Carlsbad, CA, USA) and cytokines, chemokines and immune cell marker TNF-α, IL-6, CCL2, CCL5 and CD68 (Qiagen GmbH, Hilden, Germany) and IL-1β (Forward: AATGAAAGACGGCACACCCA, Reverse: TGCTTGTGAGGTGCTGATGT; Invitrogen, Carlsbad, CA, USA) was used. Expression levels of target genes were normalized to the target gene *Gapdh* (Qiagen GmbH, Hilden, Germany), and presented as 2^−ΔΔCT^, where ΔCT = CT_target gene_ − CT_reference gene_ and ΔΔCT = ΔCT_sample_ − ΔCT_average control group_. The values for each sample were then normalized to total cDNA in the sample, measured with the QUANT-IT™ OLIGREEN ssDNA assay kit (Invitrogen, Carlsbad, CA, USA), according to the manufacturer’s instructions [29]. The non-injured hemisphere of the saline group was used as the control.

### 2.9. Immunohistochemistry

Brains were immersed in ethanol (70%) for 1.5 h, dehydrated and embedded in paraffin. Embedded brains were sectioned on a microtome in a caudal-rostral direction and 8-µm coronal sections collected on Superfrost Plus slides (VWR international, Radnor, PA, USA) at 3 levels with 400 µm between the first section of each level.

### 2.10. Neuron and Microglia Staining

Sections were de-paraffinized in a graded series of xylene followed by re-hydration in decreasing concentrations of ethanol and boiled in citrate buffer (pH 6) for 10 min for antigen retrieval. Peroxidases were blocked by H_2_O_2_ (3%) treatment for 10 min. To block unspecific binding, sections were treated with 3% horse serum (for MAP2 staining) or 4% goat serum with 0.1% Triton-X100 (for Iba-1 staining) for 30 min. Sections were then incubated overnight at 4 °C with primary antibody: microtubulin-associated protein 2 (MAP2; clone HM-2, 1:1000; Sigma-Aldrich, St. Louis, MS, USA) or ionized calcium-binding adapter molecule 1 (Iba-1; 1:2000, Cat# 019-19741; Fujifilm Wako Chemicals U.S.A. Corporation, Richmond, VA, USA), and then incubated in corresponding biotinylated secondary antibodies (Vector Laboratories, Burlingame, CA, USA) for 60 min at room temperature.

After incubating sections with Vectastain ABC Elite (Vector Laboratories, Burlingame, CA, USA), they were developed with 0.5 mg/mL of 3,3-diaminobenzidine enhanced with 15 mg/mL ammonium nickel sulfate, 2 mg/mL β-D-glucose, 0.4 mg/mL ammonium chloride and 0.01 mg/mL β -glucose oxidase. Sections were then de-hydrated in a graded series of ethanol and xylene and mounted using Pertex mounting medium (Histolab, Askim Sweden). All chemicals were purchased from Sigma-Aldrich (St. Louis, MS, USA) unless otherwise stated. 

### 2.11. Quantification of Brain Injury and Microglial Scoring

All quantifications and scoring were performed in a blinded manner. Images of sections were captured using a light microscope. MAP2-stained areas in each hemisphere were measured using ImageJ 1.51j8 (http:/imageJ.nih.gov/ij, National Institutes of Health, Bethesda, MD, USA). Tissue loss was calculated by subtracting the MAP-2 positive area of the ipsilateral hemisphere from the contralateral hemisphere and expressed as percentage tissue loss of the non-injured hemisphere. The volume of the tissue loss was calculated as V_injury_ = V_contra_ – V_ipsi_ where V = ΣA∙T, A is the MAP2-stained section of the hemisphere at each level and T is the distance between levels (400 µm), including the thickness of the section [23]. Microglia scoring was performed using a modified version of a previously described scoring system [30,31]. In brief, brain regions, including upper and lower cortex, pyriform cortex, hippocam-pus, midbrain, substantia nigra, thalamus and striatum, were given a score 0-4 based on microglial phenotype and accumulation (Supplementary Figure S1). In brief, using a light microscope at 20×, multiple sections of selected regions (upper cortex, mid cortex, pyriform cortex, substantia nigra, midbrain, hippocampus, thalamus and striatum) were given a score between 0 and 4 based on microglial morphology and accumulation, from which a mean was calculated for each region. The overall mean score was calculated as the mean value of all regional scores in each animal.

### 2.12. Statistical Analyses and Visualization

Analysis of mRNA, protein levels and caspase-3 activity were performed in GraphPad Prism (version 9.1.1 for Windows, GraphPad Software, San Diego, CA, USA). Normal distribution was tested using Shapiro-Wilk and statistical test chosen accordingly. The variance homogeneity was examined with Levene’s test. RNA and protein measurements 24 h after HI data were analyzed using one-way ANOVA followed by Sidak’s multiple comparison test. Analysis of tissue loss, microglial activation, animal weights and behavior was performed using SPSS software (IBM SPSS Statistics for Windows, version 26; IBM, Armonk, NY, USA). Measurements of tissue loss, microglial activation, and weights were analyzed with two-way ANOVA with experimental day as nuisance factor. For analysis of behavioral data, negative geotaxis time was compared using Mann-Whitney U test for two independent groups (saline and PHA) for each day (24 and 48 h after HI), and righting direction scores were analyzed with Pearson’s chi-squared test. For analysis of alteration in behavioral data between experimental days (24 and 48 h after HI) within each group, two-tailed paired t-test was used. All graphs were generated using GraphPad Prism (version 9.1.1 for Windows, GraphPad Software, San Diego, CA, USA). The number of mice used per experiment and other specific statistical details are stated in the figure legends. *p* < 0.05 was considered significant. Graphic illustrations were created using BioRender.com.

## 3. Results

### 3.1. Activation of α7nAChR Does Not Alter α7nAChR mRNA Expression in the Brain

Using qPCR, the gene coding for α7nAChR, *Chrna7,* was measured 24 h after HI to investigate whether hypoxia ischemia (HI) and/or agonist stimulation would affect expression of the receptor (Figure 1a). There was no difference in α7nAChR expression in the injured hemisphere compared with the non-injured hemisphere in either of the groups 24 h after HI. Furthermore, α7nAChR agonist PHA 568487 did not alter gene expression of the α7nAChR (Figure 1b).

### 3.2. Activation of α7nAChR Decreases Gene Expression of CCL2, CCL5 and IL-6 24 h in the Brain 24 h after Hypoxia Ischemia in Male Pups

To investigate the acute effect of α7nAChR activation on inflammation in neonatal brain injury, mRNA expression of common pro-inflammatory cytokines was measured in the brain 24 h after HI. HI injury per se increased the expression of CCL2/MCP-1, CCL5/RANTES, IL-6, TNF-α and microglia/macrophage marker CD68 in both male and female mice, whereas expression of IL-1β was not significantly altered (Figure 1c). In male, but not female pups, mRNA expression of CCL2, CCL5 and IL-6 was significantly down-regulated in the injured hemisphere in pups receiving α7nAChR agonist PHA 568487 compared with controls receiving saline (Figure 1c).

### 3.3. TNFα, Caspase-3 Activity and Tissue Loss Is Not Altered by α7nAChR Agonist Treatment at 24 h

To further investigate inflammation and apoptosis, TNFα protein levels and caspase-3 activity were analyzed in the brain 24 h after HI. The male PHA group and the female saline group displayed increased TNFα protein levels in the injured hemisphere compared with the non-injured side. However, there were no significant differences between the treatment groups (Figure 1d). HI significantly increased caspase-3 activity in male mice. A similar pattern was seen in female mice, but did not reach significance. Activation of α7nAChR did not affect caspase-3 activity in either sex (Figure 1e).

### 3.4. α7nAChR Activation Does Not Affect Tissue Loss or Microglial Activation in Neonatal Hypoxic Ischemic Brain Injury

To investigate the effect of α7nAChR activation on tissue loss, the injured area was quantified in sections from brains collected 24 h and 7 days after HI, stained with the neuron dendrite marker MAP2 (Figure 2a). There was no difference in tissue loss (%) between groups treated with α7nAChR agonist PHA 568487 and saline controls in total injured volume (Figure 2b) or in the three analyzed levels in either sex, not at 24 h or after 7 days (Figure 2c,d). To further evaluate the effect of α7nAChR activation on inflammation after HI, microglial activation was scored in Iba1-stained sections of the brain collected 7 days after HI. There was no difference in microglial activation in any of the analyzed regions (cortex, pyriform cortex, substantia nigra, hippocampus, midbrain, thalamus and striatum) between pups receiving α7nAChR agonist and saline controls (Figure 2e). 

### 3.5. Activation of α7nAChR Does Not Affect Motor Function or Weight Gain after HI

Negative geotaxis and surface righting were used as behavioral assessments to test the effect of α7nAChR treatment on motor function. There was no difference in righting time in the negative geotaxis between pups receiving α7nAChR agonist and pups receiving saline in either of the sexes 24 or 48 h after HI (Figure 2f). Furthermore, there was no difference in surface righting time in the negative geotaxis between the treatment groups in any of the sexes (Figure 2f). No difference in weight gain was found between any of the groups in the 7 days following HI (data not shown).

### 3.6. Knock-Out of α7nAChR Does Not Affect Tissue Loss in Neonatal Hypoxic-Ischemic Brain Injury

To further elucidate the role of α7nAChR in neonatal brain injury, tissue loss was measured in the brain in α7nAChR knock-outs (KO) and wild-type (WT) littermates following HI (Figure 3a). Knock-out of α7nAChR did not affect tissue loss volume (Figure 3c) or areal tissue loss at individual coronal levels (Figure 3d) in either sex.

## 4. Discussion

In the present study, we investigated the effect of α7nAChR stimulation on inflammatory responses and brain injury in neonatal mice. Agonist treatment with PHA 568487 decreased the mRNA expression of cerebral CCL2, CCL5 and IL-6 24 h after HI in male, but not female, pups. However, α7nAChR stimulation did not change caspase-3 activity 24 h after HI nor have an effect on cerebral tissue loss or microglial activation 7 days after HI, independent of sex. Furthermore, HI per se, nor agonist treatment, altered mRNA expression of α7nAChR in the brain at the 24 h timepoint.

In the periphery, the α7nAChR mediates its immune modulation mostly via expression on monocytes and macrophages [14,32]. The most profound effects of α7nAChR activation are dampening cytokine release and production [14], but it can also effect phagocytosis [33].Within the CNS, α7nAChR is expressed by neurons and astrocytes. In neurons, α7nAChR has an important role for regulating synaptic plasticity [34], and in astrocytes, α7nAChR is suggested to protect against oxidative stress [35] as well as to dampen inflammation [36]. Being the brain macrophage, microglia express α7nAChR [28]. Agonist stimulation in primary microglia cultures have shown to decrease cytokine production [28,37,38], highlighting an immune-modulatory role of α7nAChR also in microglial cells.

Inflammation plays a central role in the developing brain, both for the normal brain development but also during pathological conditions, such as cerebral HI [39]. During HI and other pathological conditions, microglia become activated, expressing co-stimulatory markers [10,40] and secreting pro-inflammatory cytokines, such as TNF-α, IL-6 and IL-1β [10,41]. These cytokines are found in CSF in HIE infants, and IL-1β levels in CSF correspond to HIE severity in these babies [42]. Given the central roles of IL-6, TNF-α and IL-1β for microglia activation and the immune responses in the brain, we investigated the expression of these genes and protein levels of TNF-α 24 h after HI. The HI insult, per se, increased gene expression of CCL2/MCP-1, CCL5/RANTES, IL-6, TNF-α and microglia/macrophage marker CD68 in the injured ipsilateral hemisphere regardless of treatment; however, the effect was most profound in male mice. Several publications have highlighted the temporal expression pattern of these cytokines, with its highest expression around 6–12 h after HI, thereafter returning to levels in parity with the contralateral side over the following 14 days [43,44,45,46]. Even though the increase in cytokine expression is not as dramatic at 24 h as reported for earlier time points [44], our data confirm that these cytokines are regulated in HI-induced brain injury. 

Interestingly, CCL2/MCP-1, CCL5/RANTES and IL-6 gene expression were significantly decreased by PHA treatment 24 h after HI in male mice. Both CCL2/MCP-1 and CCL5/RANTES are upregulated during HI [7] and participate in the recruitment of immune cells into the CNS after injury [47,48,49]. Thus, the decreased expression of CCL2/MCP-1 and CCL5/RANTES indicate a possible effect of α7nAChR stimulation on cell recruitment. However, when investigating the CD68 expression as a marker of peripheral monocytes/macrophages, we did not detect any difference of α7nAChR stimulation. Indeed, CD68 can be expressed by both microglia and peripheral monocytes/macrophages, and it is possible that the 24 h time point is too early for detecting an effect on peripheral involvement.

In contrast to TNF-α and IL-1β, IL-6 is considered a pleiotropic cytokine, with dual roles in inflammation and neurogenesis [50,51]. While it activates microglia cells and acts pro-inflammatory during the acute phase, it may also act as a neurotrophic mediator, promoting neuronal survival after injury [50,52]. Given the relatively short time frame in the study, it is more likely an acute anti-inflammatory effect we observed in male mice by PHA treatment. 

Apoptosis is an important regulator of normal brain development, tightly regulating the formation of the CNS [53]. However, insults such as HI may trigger several types of cell death, including both apoptosis and necrosis [53]. We investigated apoptosis by measuring caspase-3 activation and found HI to increase caspase-3 activity in both male and female mice to a similar level. In contrast, some studies have suggested females to mainly regulate cell death through caspase-dependent pathways [54], while cell death in male mice has been associated with inflammation-driven necrosis [55,56,57].

In addition to the differences in cell-death pathways between male and female mice, there is an increasing number of studies highlighting sex differences in neonatal brain injury development. Although this data is not conclusive, there seems to be an increased susceptibility for male mice to develop larger brain injury compared to female mice [29]. This is also seen in humans, e.g., in children born extremely pre-term, cognitive function is poorer in boys compared with girls [58]. Furthermore, α7nAChR have been reported to display a sexually dimorphic response when investigating hippocampal neurogenesis [59]. Given this background, we included both sexes when investigating the potential role of α7nAChR stimulation in neonatal brain injury. However, we did not find any support for α7nAChR to mediate sex-driven effects on brain injury, microglia activation or motor function. 

Stimulation of α7nAChR in adult brain injury models have been tested with PHA 568487 as well as other agonists/PAMs, such as PHA 543613 and PNU 282987, where several studies demonstrate a beneficial effect on injury development [15,16,18,19,20,21], while a previous study in our lab showed lack of effect of AR-R17779 in transient MCAO [31]. The agonist chosen for this study, PHA 568487, has shown promising results in models of adult brain injury [20]. Nevertheless, in the current study, we do not find any evidence for PHA 568487 to influence brain injury. The decrease in IL-6 and CCL2 in males by agonist treatment 24 h after HI indicates an immunomodulatory effect; however, it is insufficient to influence motor function or injury outcome. Interestingly, in one of the studies of adult stroke [18], the beneficial effect of agonist treatment was independent of an effect of inflammation; hence, other mechanisms of neuroprotection [19] may contribute to the positive effect. Of note, the mechanisms in adult and neonatal brain injury differ [60], and treatments that show promising results in adults may not have an effect in the developing brain; in fact, they can even display an opposite response [61]. Indeed, in a study by Laudenbach et al. [17] using an excitotoxic brain injury model, treatment with the α7nAChR agonist GTS-21 worsened brain injury, whereas it was decreased in α7nAChR knock-out mice. Although GTS-21 displays inhibitory effects on another nicotinic receptor, α4β2nAChR [62,63], and can have anti-inflammatory effects independent of α7nAChR [64], the usage of α7nAChR knock-out mice clearly demonstrates a beneficial effect of α7nAChR blockade in neonatal excitatory brain injury on PND4. To fully elucidate the role of α7nAChR in neonatal brain injury, we also investigated brain injury in mice lacking α7nAChR and their littermate controls. However, in the HI model, we do not see any differences in brain injury between α7nAChR KO and littermate controls in males or females. 

The gene expression of the α7nAChR (*Chrna7*) is widespread throughout the brain, with its highest expression in the hippocampus and cortex [31]. We have previously seen that α7nAChR gene expression (*Chrna7*) in the injured hemisphere is not altered 24 h after HI, compared to the contralateral hemisphere, whereas 3 days after HI, *Chrna7* expression is decreased [28]. In the current study, we can confirm our previous findings, i.e., that gene expression of α7nAChR is not changed 24 h post HI between the injured and non-injured hemisphere, and that the expression is not altered by treatment with α7nAChR agonist PHA 568487.

## 5. Conclusions

Despite a decrease in gene expression of inflammatory markers CCL2/MCP-1, CCL5/RANTES and IL-6 in α7nAChR agonist-treated male mice 24 hours after neonatal brain injury, we did not find any evidence for α7nAChR to influence overall injury outcome or microglia activation in either male or female pups, neither by stimulation nor knock-out of α7nAChR. Thus, in neonatal HI, α7nAChR does not seem to play a significant role in brain injury processes. 

## Data Availability

All data are included within the main article and the supplementary file.

## Supplementary Materials

The following supporting information can be downloaded at: https://www.mdpi.com/article/10.3390/biomedicines10082023/s1, Figure S1: Microglia scoring method.

## References

[1] Douglas-Escobar M., Weiss M.D. (2015). Hypoxic-ischemic encephalopathy: A review for the clinician. JAMA Pediatr..

[2] Perez A., Ritter S., Brotschi B., Werner H., Caflisch J., Martin E., Latal B. (2013). Long-term neurodevelopmental outcome with hypoxic-ischemic encephalopathy. J. Pediatr..

[3] Simbruner G., Mittal R.A., Rohlmann F., Muche R., Participants N.T. (2010). Systemic Hypothermia After Neonatal Encephalopathy: Outcomes of neo.nEURO.network RCT. Pediatrics.

[4] Zhou W.-H., Cheng G.-Q., Shao X.-M., Liu X.-Z., Shan R.-B., Zhuang D.-Y., Zhou C.-L., Du L.-Z., Cao Y., Yang Q. (2010). Selective Head Cooling with Mild Systemic Hypothermia after Neonatal Hypoxic-Ischemic Encephalopathy: A Multicenter Randomized Controlled Trial in China. J. Pediatr..

[5] Edwards A.D., Brocklehurst P., Gunn A., Halliday H., Juszczak E., Levene M., Strohm B., Thoresen M., Whitelaw A., Azzopardi D. (2010). Neurological outcomes at 18 months of age after moderate hypothermia for perinatal hypoxic ischaemic encephalopathy: Synthesis and meta-analysis of trial data. BMJ.

[6] Dammann O., Leviton A. (2004). Inflammatory brain damage in preterm newborns—Dry numbers, wet lab, and causal inferences. Early Hum. Dev..

[7] Hedtjarn M., Mallard C., Hagberg H. (2004). Inflammatory gene profiling in the developing mouse brain after hypoxia-ischemia. J. Cereb. Blood Flow Metab..

[8] Vexler Z.S., Tang X.N., Yenari M.A. (2006). Inflammation in adult and neonatal stroke. Clin. Neurosci. Res..

[9] Concepcion K.R., Zhang L. (2018). Corticosteroids and perinatal hypoxic-ischemic brain injury. Drug Discov. Today.

[10] Aloisi F. (2001). Immune function of microglia. Glia.

[11] Albuquerque E.X., Alkondon M., Pereira E.F.R., Castro N.G., Schrattenholz A., Barbosa C.T.F., Bonfante-Cabarcas R., Aracava Y., Eisenberg H.M., Maelicke A. (1997). Properties of neuronal nicotinic acetylcholine receptors: Pharmacological characterization and mod-ulation of synaptic function. J. Pharmacol. Exp. Ther..

[12] Kihara T., Shimohama S., Sawada H., Honda K., Nakamizo T., Shibasaki H., Toshiaki K., Akinori A. (2001). alpha 7 nicotinic receptor transduces signals to phosphatidylinositol 3-kinase to block A beta-amyloid-induced neurotoxicity. J. Biol. Chem..

[13] Sinkus M.L., Graw S., Freedman R., Ross R.G., Lester H.A., Leonard S. (2015). The human CHRNA7 and CHRFAM7A genes: A review of the genetics, regulation, and function. Neuropharmacology.

[14] Wang H., Yu M., Ochani M., Amella C.A., Tanovic M., Susarla S., Li J.H., Wang H., Yang H., Ulloa L. (2003). Nicotinic acetylcholine receptor alpha7 subunit is an essential regulator of inflammation. Nature.

[15] Jiang T., Wu M., Zhang Z., Yan C., Ma Z., He S., Wei Y., Pu K., Wang Q. (2019). Electroacupuncture attenuated cerebral ischemic injury and neuroinflammation through al-pha7nAChR-mediated inhibition of NLRP3 inflammasome in stroke rats. Mol. Med..

[16] Krafft P.R., McBride D., Rolland W.B., Lekic T., Flores J.J., Zhang J.H. (2017). *α*7 Nicotinic Acetylcholine Receptor Stimulation Attenuates Neuroinflammation through JAK2-STAT3 Activation in Murine Models of Intracerebral Hemorrhage. BioMed Res. Int..

[17] Laudenbach V., Medja F., Zoli M., Rossi F.M., Evrard P., Changeux J.-P., Gressens P. (2002). Selective activation of central subtypes of the nicotinic acetylcholine receptor has opposite effects on neonatal excitotoxic brain injuries. FASEB J..

[18] Colas L., Domercq M., Ramos-Cabrer P., Palma A., Gómez-Vallejo V., Padro D., Plaza-García S., Pulagam K.R., Higuchi M., Matute C. (2018). In vivo imaging of Alpha7 nicotinic receptors as a novel method to monitor neuroinflammation after cerebral ischemia. Glia.

[19] Wang J., Lu Z., Fu X., Zhang D., Yu L., Li N., Gao Y., Liu X., Yin C., Ke J. (2017). Alpha-7 Nicotinic Receptor Signaling Pathway Participates in the Neurogenesis Induced by ChAT-Positive Neurons in the Subventricular Zone. Transl. Stroke Res..

[20] Han Z., Li L., Wang L., Degos V., Maze M., Su H. (2014). Alpha-7 nicotinic acetylcholine receptor agonist treatment reduces neuroinflammation, oxidative stress, and brain injury in mice with ischemic stroke and bone fracture. J. Neurochem..

[21] Krafft P.R., Altay O., Rolland W.B., Duris K., Lekic T., Tang J., Zhang J.H. (2012). alpha7 nicotinic acetylcholine receptor agonism confers neuroprotection through GSK-3beta inhibition in a mouse model of intracerebral hemorrhage. Stroke.

[22] Rice J.E., Vannucci R.C., Brierley J.B. (1981). The influence of immaturity on hypoxic-ischemic brain damage in the rat. Ann. Neurol..

[23] Svedin P., Hagberg H., Sävman K., Zhu C., Mallard C. (2007). Matrix Metalloproteinase-9 Gene Knock-out Protects the Immature Brain after Cerebral Hypoxia–Ischemia. J. Neurosci..

[24] Semple B.D., Blomgren K., Gimlin K., Ferriero D.M., Noble-Haeusslein L.J. (2013). Brain development in rodents and humans: Identifying benchmarks of maturation and vulnerability to injury across species. Prog. Neurobiol..

[25] Walker D.P., Wishka D.G., Piotrowski D.W., Jia S., Reitz S.C., Yates K.M., Myers J.K., Vetman T.N., Margolis B.J., Jacobsen E.J. (2006). Design, synthesis, structure-activity relationship, and in vivo activity of azabicyclic aryl amides as alpha7 nicotinic acetylcholine receptor agonists. Bioorg. Med. Chem..

[26] Feather-Schussler D.N., Ferguson T.S. (2016). A Battery of Motor Tests in a Neonatal Mouse Model of Cerebral Palsy. J. Vis. Exp..

[27] Li T., Li K., Zhang S., Wang Y., Xu Y., Cronin S.J.F., Sun Y., Zhang Y., Xie C., Rodriguez J.I. (2020). Overexpression of apoptosis inducing factor aggravates hypoxic-ischemic brain injury in neonatal mice. Cell Death Dis..

[28] Hua S., Ek C.J., Mallard C., Johansson M.E. (2014). Perinatal hypoxia-ischemia reduces alpha 7 nicotinic receptor expression and selective alpha 7 nicotinic receptor stimulation suppresses inflammation and promotes microglial Mox phenotype. Biomed. Res. Int..

[29] Gravina G., Svedin P., Ardalan M., Levy O., Ek C.J., Mallard C., Lai J. (2020). Staphylococcus epidermidis Sensitizes Perinatal Hypoxic-Ischemic Brain Injury in Male but Not Female Mice. Front. Immunol..

[30] Rocha-Ferreira E., Poupon L., Zelco A., Leverin A.L., Nair S., Jonsdotter A., Carlsson Y., Thornton C., Hagberg H., Rahim A.A. (2018). Neuroprotective exendin-4 enhances hypothermia therapy in a model of hypoxic-ischaemic enceph-alopathy. Brain.

[31] Hammarlund M.E., Darsalia V., Mjörnstedt F., Pattanaik B., Mallard C., Rocha-Ferreira E., Patrone C., Johansson M.E. (2021). The selective alpha7 nicotinic acetylcholine receptor agonist AR-R17779 does not affect ischemia–reperfusion brain injury in mice. Biosci. Rep..

[32] Johansson M.E., Ulleryd M.A., Bernardi A., Lundberg A.M., Andersson A., Folkersen L., Fogelstrand L., Islander U., Yan Z.-Q., Hansson G.K. (2014). α7 Nicotinic Acetylcholine Receptor Is Expressed in Human Atherosclerosis and Inhibits Disease in Mice—Brief Report. Arter. Thromb. Vasc. Biol..

[33] Ulleryd M.A., Mjörnstedt F., Panagaki D., Yang L.J., Engevall K., Gutiérrez S., Wang Y., Gan L.-M., Nilsson H., Michaëlsson E. (2019). Stimulation of alpha 7 nicotinic acetylcholine receptor (alpha7nAChR) inhibits atherosclerosis via im-munomodulatory effects on myeloid cells. Atherosclerosis.

[34] Xu Z.-Q., Zhang W.-J., Su D.-F., Zhang G.-Q., Miao C.-Y. (2021). Cellular responses and functions of α7 nicotinic acetylcholine receptor activation in the brain: A narrative review. Ann. Transl. Med..

[35] Liu Y., Zeng X., Hui Y., Zhu C., Wu J., Taylor D.H., Ji J., Fan W., Huang Z., Hu J. (2015). Activation of α7 nicotinic acetylcholine receptors protects astrocytes against oxidative stress-induced apoptosis: Implications for Parkinson’s disease. Neuropharmacology.

[36] Revathikumar P., Bergqvist F., Gopalakrishnan S., Korotkova M., Jakobsson P.-J., Lampa J., Le Maître E. (2016). Immunomodulatory effects of nicotine on interleukin 1β activated human astrocytes and the role of cyclooxygenase 2 in the underlying mechanism. J. Neuroinflamm..

[37] Shytle R.D., Mori T., Townsend K., Vendrame M., Sun N., Zeng J., Ehrhart J., Silver A.A., Sanberg P.R., Tan J. (2004). Cholinergic modulation of microglial activation by α7 nicotinic receptors. J. Neurochem..

[38] Suzuki T., Hide I., Matsubara A., Hama C., Harada K., Miyano K., Andrä M., Matsubayashi H., Sakai N., Kohsaka S. (2006). Microglial α7 nicotinic acetylcholine receptors drive a phospholipase C/IP3 pathway and modulate the cell activation toward a neuroprotective role. J. Neurosci. Res..

[39] Hagberg H., Mallard C., Ferriero D.M., Vannucci S.J., Levison S.W., Vexler Z.S., Gressens P. (2015). The role of inflammation in perinatal brain injury. Nat. Rev. Neurol..

[40] Rocha-Ferreira E., Vincent A., Bright S., Peebles D.M., Hristova M. (2018). The duration of hypothermia affects short-term neuro-protection in a mouse model of neonatal hypoxic ischaemic injury. PLoS ONE.

[41] Smith J.A., Das A., Ray S.K., Banik N.L. (2012). Role of pro-inflammatory cytokines released from microglia in neurodegenerative diseases. Brain Res. Bull..

[42] Aly H., Khashaba M.T., El-Ayouty M., El-Sayed O., Hasanein B.M. (2006). IL-1β, IL-6 and TNF-α and outcomes of neonatal hypoxic ischemic encephalopathy. Brain Dev..

[43] Szaflarski J., Burtrum D., Silverstein F.S. (1995). Cerebral Hypoxia-Ischemia Stimulates Cytokine Gene Expression in Perinatal Rats. Stroke.

[44] Bona E., Andersson A.-L., Blomgren K., Gilland E., Puka-Sundvall M., Gustafson K., Hagberg H. (1999). Chemokine and Inflammatory Cell Response to Hypoxia-Ischemia in Immature Rats. Pediatr. Res..

[45] Shrivastava K., Llovera G., Recasens M., Chertoff M., Giménez-Llort L., Gonzalez B., Acarin L. (2013). Temporal Expression of Cytokines and Signal Transducer and Activator of Transcription Factor 3 Acti-vation after Neonatal Hypoxia/Ischemia in Mice. Dev. Neurosci..

[46] Hagberg H., Gilland E., Bona E., Hanson L.-Å., Hahn-Zoric M., Blennow M., Holst M., McRae A., Söder O. (1996). Enhanced Expression of Interleukin (IL)-1 and IL-6 Messenger RNA and Bioactive Protein after Hypox-ia-Ischemia in Neonatal Rats. Pediatr. Res..

[47] Andres R.H., Choi R., Pendharkar A.V., Gaeta X., Wang N., Nathan J.K., Chua J.Y., Lee S.W., Palmer T.D., Steinberg G.K. (2011). The CCR2/CCL2 interaction mediates the transendothelial recruitment of intravascularly delivered neural stem cells to the ischemic brain. Stroke.

[48] Appay V., Rowland-Jones S.L. (2001). RANTES: A versatile and controversial chemokine. Trends Immunol..

[49] Hughes P.M., Allegrini P.R., Rudin M., Perry V.H., Mir A.K., Wiessner C. (2002). Monocyte Chemoattractant Protein-1 Deficiency is Protective in a Murine Stroke Model. J. Cereb. Blood Flow Metab..

[50] Kölliker-Frers R., Udovin L., Otero-Losada M., Kobiec T., Herrera M.I., Palacios J., Razzitte G., Capani F. (2021). Neuroinflammation: An Integrating Overview of Reactive-Neuroimmune Cell Interactions in Health and Disease. Mediat. Inflamm..

[51] Erta M., Quintana A., Hidalgo J. (2012). Interleukin-6, a Major Cytokine in the Central Nervous System. Int. J. Biol. Sci..

[52] Suzuki S., Tanaka K., Suzuki N. (2009). Ambivalent Aspects of Interleukin-6 in Cerebral Ischemia: Inflammatory versus Neurotrophic Aspects. J. Cereb. Blood Flow Metab..

[53] Thornton C., Leaw B., Mallard C., Nair S., Jinnai M., Hagberg H. (2017). Cell Death in the Developing Brain after Hypoxia-Ischemia. Front. Cell. Neurosci..

[54] Zhu C., Xu F., Wang X., Shibata M., Uchiyama Y., Blomgren K., Hagberg H. (2006). Different apoptotic mechanisms are activated in male and female brains after neonatal hypoxia-ischaemia. J. Neurochem..

[55] Chavez-Valdez R., Mottahedin A., Stridh L., Yellowhair T.R., Jantzie L.L., Northington F.J., Mallard C. (2019). Evidence for Sexual Dimorphism in the Response to TLR3 Activation in the Developing Neonatal Mouse Brain: A Pilot Study. Front. Physiol..

[56] Hurn P.D., Vannucci S.J., Hagberg H. (2005). Adult or Perinatal Brain Injury. Stroke.

[57] Cheng J., Hurn P.D. (2010). Sex shapes experimental ischemic brain injury. Steroids.

[58] Skiöld B., Alexandrou G., Padilla N., Blennow M., Vollmer B., Ådén U. (2014). Sex Differences in Outcome and Associations with Neonatal Brain Morphology in Extremely Preterm Children. J. Pediatr..

[59] Otto S.L., Yakel J.L. (2019). The α7 nicotinic acetylcholine receptors regulate hippocampal adult-neurogenesis in a sexually dimorphic fashion. Anat. Embryol..

[60] Vexler Z.S., Yenari M.A. (2009). Does inflammation after stroke affect the developing brain differently than adult brain?. Dev. Neurosci..

[61] Doverhag C., Hedtjärn M., Poirier F., Mallard C., Hagberg H., Karlsson A., Sävman K. (2010). Galectin-3 contributes to neonatal hypoxic–ischemic brain injury. Neurobiol. Dis..

[62] Hunter B.E., de Fiebre C.M., Papke R.L., Kem W.R., Meyer E.M. (1994). A novel nicotinic agonist facilitates induction of long-term potentiation in the rat hippocampus. Neurosci. Lett..

[63] Meyer E.M., Kuryatov A., Gerzanich V., Lindstrom J., Papke R.L. (1998). Analysis of 3-(4-hydroxy, 2-Methoxybenzylidene)anabaseine selectivity and activity at human and rat alpha-7 nicotinic receptors. J. Pharmacol. Exp. Ther..

[64] Garg B.K., Loring R.H. (2019). GTS-21 has cell-specific anti-inflammatory effects independent of α7 nicotinic acetylcholine receptors. PLoS ONE.

