# Supplementary material for: The Alpha 7 Nicotinic Acetylcholine Receptor Does Not Affect Neonatal Brain Injury
Source: Biomedicines. 2022 Aug 19;10(8):2023. doi: 10.3390/biomedicines10082023 (PMC9405910; doi:10.3390/biomedicines10082023)
Supplement: Supplementary file 1 [file biomedicines-10-02023-s001.zip › biomedicines-1835920-supplementary.pdf]

Supplementary figure S1

a

Score	Iba-1 immunoreactivity
**0**	No microglial activation
**1**	Focal activation
**2**	Mild diffuse activation, occasional amoeboid microglia
**3**	Widespread activation, predominant amoeboid microglia
**4**	Tissue loss

b

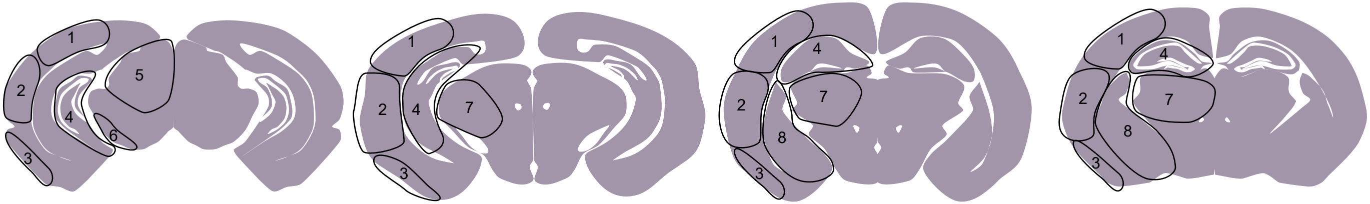

Supplementary Figure S1. (**a**) Definition of microglial scoring (**b**) Pictures of coronal brain sections illustrating scored regions; 1: cortex 1 (CTX1); 2: cortex 2 (CTX2); 3: pyriform cortex (PYR); 4: hippocampus (HIP); 5: midbrain (MB); 6: substantia nigra (SN); 7: thalamus (THL); 8: striatum (STR)
